# Efficient signal sequence of mRNA vaccines enhances the antigen expression to expand the immune protection against viral infection

**DOI:** 10.1186/s12951-024-02488-3

**Published:** 2024-05-28

**Authors:** Yupei Zhang, Songhui Zhai, Hai Huang, Shugang Qin, Min Sun, Yuting Chen, Xing Lan, Guohong Li, Zhiying Huang, Denggang Wang, Yaoyao Luo, Wen Xiao, Hao Li, Xi He, Meiwan Chen, Xingchen Peng, Xiangrong Song

**Affiliations:** 1 Department of Critical Care Medicine, Frontiers Science Center for Disease-related Molecular Network, State Key Laboratory of Biotherapy and Cancer Center, West China Hospital, Sichuan University, Chengdu, 610041 China; 2 Department of Pediatrics, West China Second University Hospital, Sichuan University, Chengdu, Sichuan 610041 China; 3 State Key Laboratory of Quality Research in Chinese Medicine, Institute of Chinese Medical Sciences, University of Macau, Macau, China

**Keywords:** Virus, mRNA vaccines, Signal sequence, SRP, Immune response

## Abstract

The signal sequence played a crucial role in the efficacy of mRNA vaccines against virus pandemic by influencing antigen translation. However, limited research had been conducted to compare and analyze the specific mechanisms involved. In this study, a novel approach was introduced by substituting the signal sequence of the mRNA antigen to enhance its immune response. Computational simulations demonstrated that various signal peptides differed in their binding capacities with the signal recognition particle (SRP) 54 M subunit, which positively correlated with antigen translation efficiency. Our data revealed that the signal sequences of tPA and IL-6-modified receptor binding domain (RBD) mRNA vaccines sequentially led to higher antigen expression and elicited more robust humoral and cellular immune protection against the SARS-CoV-2 compared to the original signal sequence. By highlighting the importance of the signal sequence, this research provided a foundational and safe approach for ongoing modifications in signal sequence-antigen design, aiming to optimize the efficacy of mRNA vaccines.

**Supplementary Information:**

The online version contains supplementary material available at 10.1186/s12951-024-02488-3.

## Introduction

Research on mRNA vaccines for preventing viral infection expanded significantly and entered clinical trials, encompassing various viruses such as human cytomegalovirus (CMV) [1], Ebola virus [2], Epstein-Barr virus (EBV) [3], rabies virus [4, 5], Zika virus [6, 7], human metapneumovirus (hMPV) [8], respiratory syncytial virus (RSV) [9], influenza virus [10, 11], and severe acute respiratory syndrome coronavirus 2 (SARS-CoV-2) [12]. Due to uncertain efficacy, researchers proposed various strategies to optimize lipid nanoparticle (LNP)-based mRNA vaccines [13, 14]. These strategies primarily involved designing new lipid carrier materials to enhance protein expression [15], introducing sequences to make the translated proteins form polymers to increase immunogenicity [16, 17], optimizing new 5’ and 3’ UTR sequences to regulate translation [18], utilizing circular RNA to avoid degradation [13], and applying self-amplifying RNA to augment quantity [19, 20]. Effective mRNA vaccine design against viruses requires specific signal sequences, without which there is no immune protective effect [21]. A previous study reported that designing a human kappa immunoglobulin signal sequence in an mRNA vaccine elicited a stronger immune response against the Ebola virus than the WT signal sequence [22]. These findings suggest that signal sequence optimization could enhance the efficacy of mRNA vaccines.

Signal sequences, also known as signal peptides, are short N-terminal secretory signals (approximately 15–25 amino acids in length) that prompt the secretion and translocation of newly synthesized proteins within the cell [23]. They mediate the targeting of nascent secretory and membrane proteins to the endoplasmic reticulum (ER) in a signal recognition particle (SRP)-dependent manner [24–26]. Subsequently, the signal peptide is cleaved by the signal peptidase on the ER luminal surface, allowing the new peptide chain to continue its extension until completion [27]. It has been reported that the signal sequences of DNA influence the expression of secretory proteins produced by in vitro cells [28–30]. Consequently, the immune response of gene vaccines might be affected by the signal sequences, as these sequences can influence antigen expression, thereby impacting B and T cell responses [31–35]. In light of this, we hypothesized that efficient signal sequences in mRNA vaccines enhance antigen expression, thereby broadening immune protection against viral infection.

**Fig. 1:**
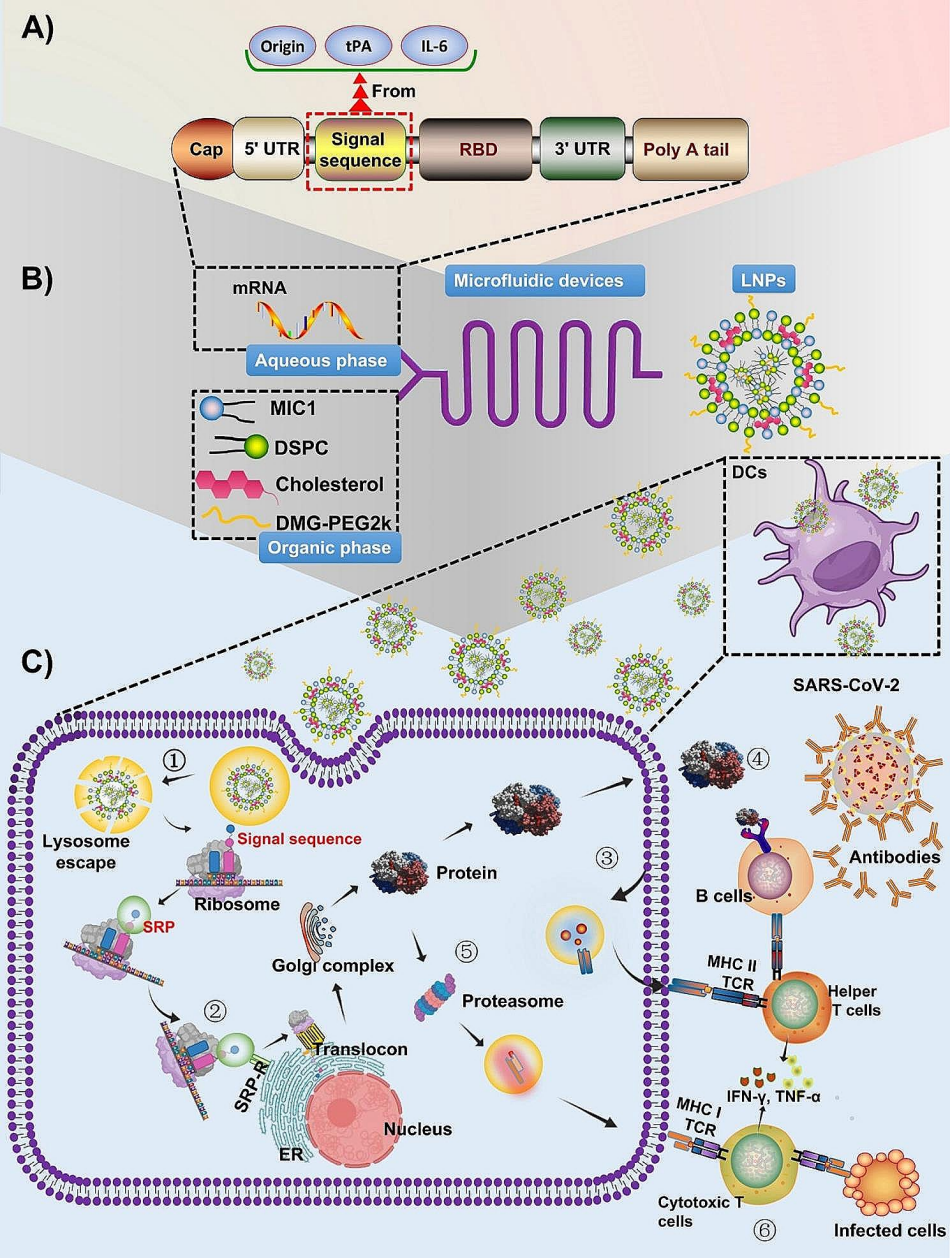
Graphical abstract depicting the mechanisms of mRNA vaccines with various signal sequences in combating viral diseases. (**A**) The mRNA sequences are designed to encode an antigen, incorporating signal sequences derived from the virus’ origin, interleukin-6 (IL-6), and tissue plasminogen activator (tPA). (**B**) Lipid nanoparticles (LNPs) are prepared utilizing a microfluidic device. (**C**) The process involves multiple steps: (1) Dendritic cells (DCs) endocytose the mRNA vaccines encoding the antigen. Subsequently, the mRNAs escape from lysosomes and was translated by ribosomes. The signal recognition particle (SRP) then bound to the translated signal sequences via the SRP54M subunit, simultaneously attaching to the ribosome, thus forming an SRP-ribosome complex. This complex temporarily halte translation. (2) The SRP recognize the SRP receptor (SRP-R) on the endoplasmic reticulum (ER) membrane, facilitating the ribosome’s anchoring to the translocon, which allow the translation to resume. The nascent peptide chain traversed the membrane, entering the ER lumen, where the signal sequences were cleaved by signal peptidase. The elongating peptide chain undergo further processing and modification within the ER and Golgi complex. (3) The secreted antigens are potentially reabsorbed by DCs, subsequently being degraded into smaller fragments within endosomes. These fragments are then presented on the cell surface to helper T (Th) cells through major histocompatibility complex (MHC) class II T cell receptors (TCRs). (4) B cells, upon receiving the initial signal from the antigen through B cell receptors (BCRs) and a secondary signal from activated Th cells, become activated and differentiated into plasma cells. These plasma cells produce antibodies to neutralize the virus. (5) Intracellular antigens are degraded into smaller fragments by proteasomes. These fragments were then presented to cytotoxic T (Tc) cells via MHC class I TCRs. (6) The activated Tc cells secrete perforin and granzyme, which targeted and eradicated infected cells by inducing apoptosis

In this study, we selected SARS-CoV-2 as the focus of our investigation. We developed and compared three distinct signal sequences from original virus, tissue plasminogen activator (tPA), and interleukin-6 (IL-6), all of which were attached to the receptor-binding domain (RBD) of mRNA vaccines. The RBD was chosen as a potential vaccine antigen due to its role in facilitating the virus’s entry into alveolar cells for replication via interaction with the angiotensin-converting enzyme 2 (ACE2) receptor [36–38]. We concurrently evaluated all three designs to assess their RBD expression levels in vitro and their immune response efficacy against SARS-CoV-2 in vivo. Our hypothesis centered on the possibility that the affinity of the signal sequences for the SRP54M subunit might influence the mRNA translation process. To explore this, we conducted computational simulations to model the binding interactions and forces between the SRP and the signal sequences. The involved process was visually depicted in Fig. 1. Through validating the significance of an effective signal sequence, our research aimed to establish a foundational framework for the ongoing development of signal sequence-modified antigen design, with the ultimate goal of enhancing mRNA vaccine effectiveness against viral infection.

## Results

### Preparation and characterization of mRNA vaccines

**Fig. 2:**
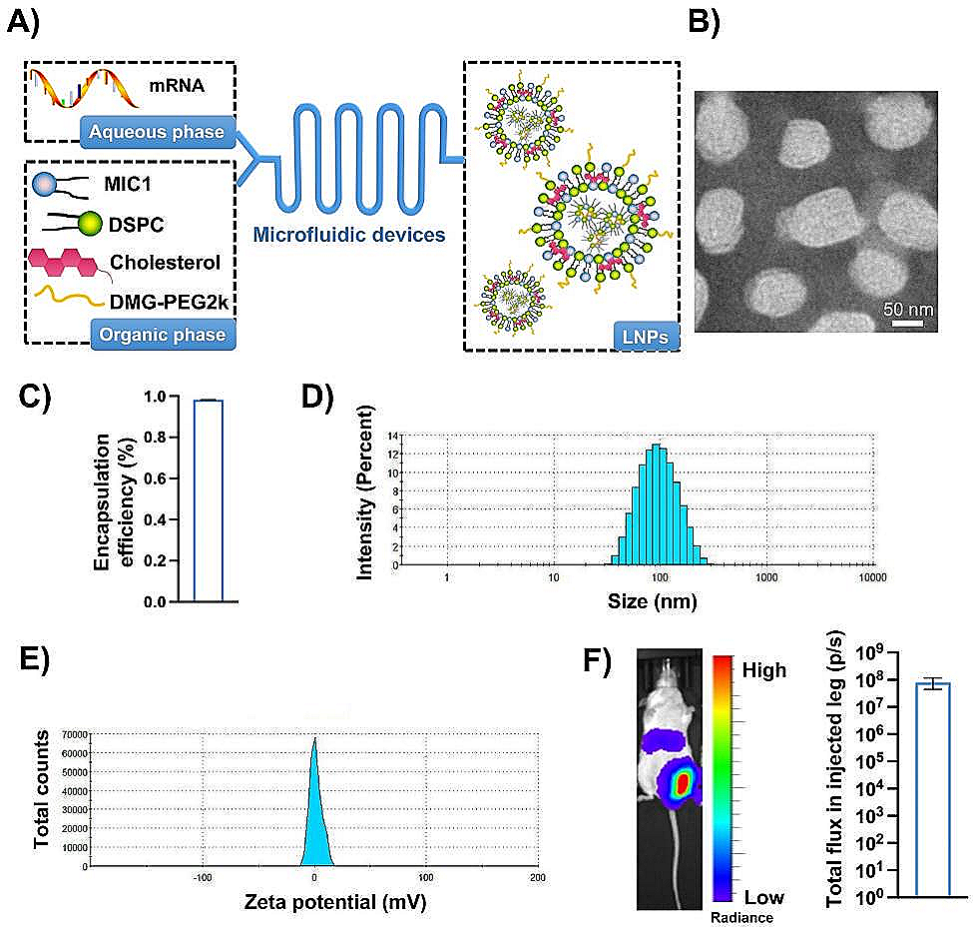
Preparation and characterization of mRNA vaccines. (**A**) Flow chart demonstrates the process employed by a microfluidic device to prepare lipid nanoparticles (LNPs) for the mRNA vaccine. (**B**) Transmission electron microscopy (TEM) image provides a detailed visualization of the LNPs. (**C**) The encapsulation efficiency of the mRNA within the LNPs is quantified. (**D**) Representative size distribution diagram showcases the range of particle sizes in the preparation. (**E**) The zeta potential of the LNPs, indicating their surface charge characteristics, is depicted in a representative diagram. (**F**) The distribution and expression levels of the mRNA vaccines in vivo were assessed following injection into the unilateral leg of mice. The results were presented as the mean ± SEM, *n* = 3

Microfluidic devices were utilized to prepare mRNA vaccines, as detailed in the Materials and methods section (Fig. 2A). Notably, the ionizable lipids MIC1, developed by our group, exhibited superior antibody titers against the virus compared to SM-102, which is already widely used in mRNA vaccines [15]. The morphology of the mRNA vaccines is depicted in Fig. 2B. The encapsulation efficiency reached 98.43% (Fig. 2C), the particle size was 100.5 nm, the PDI of the size distribution was 0.21 (Fig. 2D), and the zeta potential was measured at − 0.12 mV (Fig. 2E). In vivo expression was evaluated through bioluminescent imaging of LUC mRNA, revealing predominant expression in the injected leg with a total flux of 1.07 × 10^8^ photons per second. While other organs had no much strong expression. As well, different doses of mRNA LNPs was added to cultured 293T and DC2.4 cells to measure the cytotoxicity in Figure S1. The cell viability didn’t decrease in a dose dependent manner even in a relatively high dose, which showed good safety in vitro.

### Signal sequences with different binding affinity to SRP54M subunit modified mRNA vaccines had different antigen expression levels

**Fig. 3:**
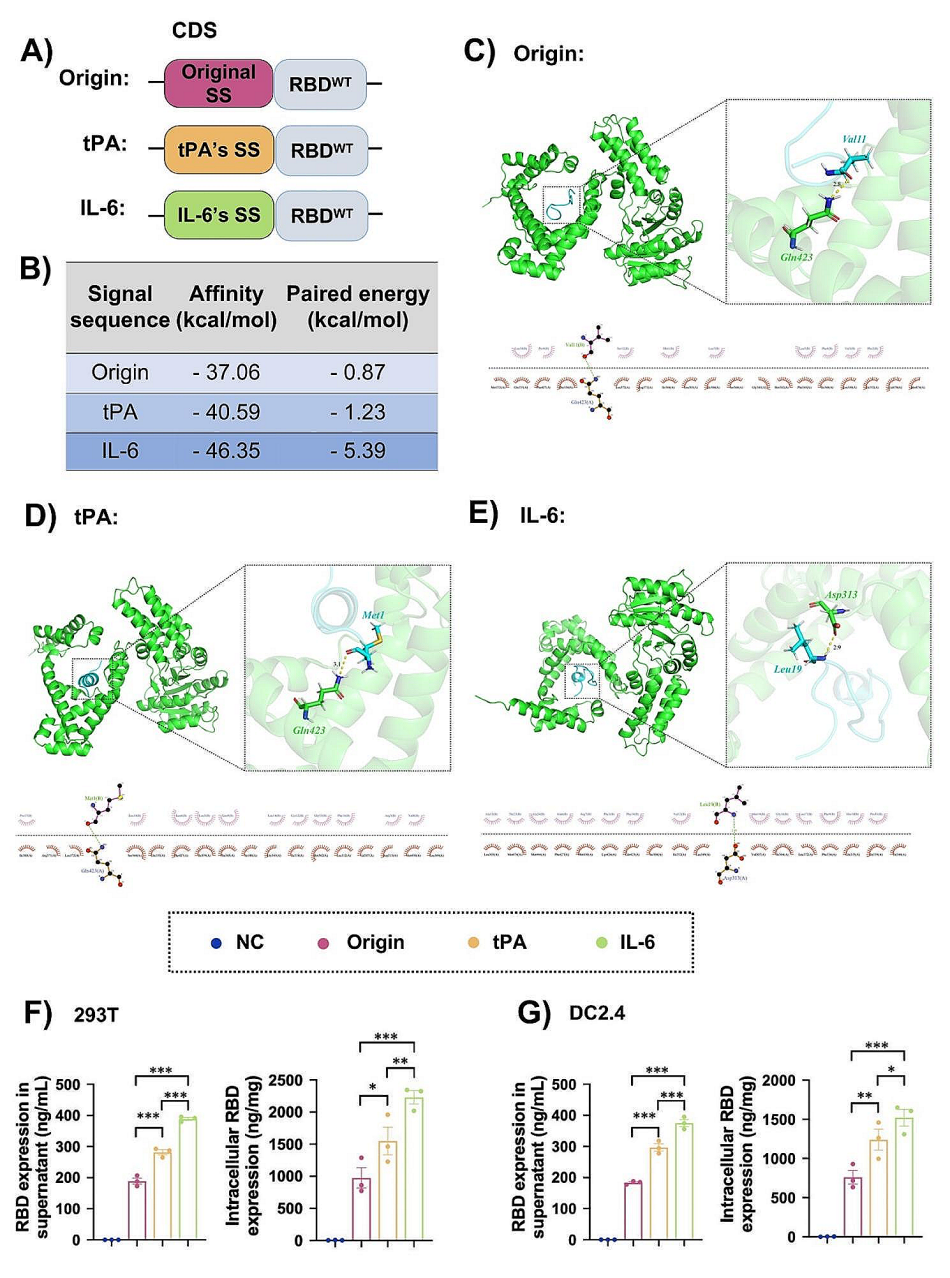
Binding affinity of different signal sequences with SRP54M subunit and antigen expression levels of mRNA vaccines modified by different signal sequences. (**A**) Coding sequences (CDSs) of mRNA with different signal sequences (SS) employed in the study. (**B**) The affinity and paired energy of signal sequences with the SRP54M domain calculated by computational simulation. 2D eyelash chart and 3D dominant binding conformation of SRP54M subunit to the signal peptides of (**C**) origin, (**D**) tPA, and (**E**) IL-6 by computational simulation. As showed in the 2D diagram, the dashed line indicated hydrogen bonding, and the number next to it indicated the hydrogen bond length. The remaining eyelashes-like residues represented hydrophobic interactions, where the eyelashes pointed towards the key residues that produced hydrophobic interactions. The 3D diagram showed the overall and local conformation of the signal peptides binding with SRP54M subunit. Intracellular and culture supernatant RBD expression levels of the different signal sequence-modified RBD^WT^ mRNA transfected (**F**) DC2.4 and (**G**) 293T cells. The results were presented as the mean ± SEM, *n* = 3. ^*^P < 0.05, ^**^P < 0.01, ^***^P < 0.001

The mRNA sequences employed in our study was showed in Fig. 3A. We used tPA and IL-6’s signal sequences to compare with original SARS-CoV-2’s signal sequence. To verify our conjecture that better recognition of signal peptide with SRP54M could cause better protein translation, computational simulation of these signal peptides with SRP54M subunit were conducted.

As shown in Fig. 3C, the main chain carboxyl group O of Val11 in the original signal peptide and the main chain amino group N-H of Gln423 residue in the SRP54M subunit formed a hydrogen bond, in a bond length of 2.8 Å. According to the analysis of the Interface energy module, the paired energy between the Val11 of original signal peptide and the Gln423 residue of SRP54 protein was − 0.87 kcal/mol (Fig. 3B), also indicating that the binding might be dominated by hydrophobic interactions.

As shown in Fig. 3D, the side chain carboxyl group O of Met1 in the tPA’s signal peptide formed a hydrogen bond with side chain amino group N-H of Gln423 residue in the SRP54M subunit, in a bond length of 3.1 Å. Analysis of the Interface energy module revealed that the paired energy between the Met1 of tPA’s signal peptide and the Gln423 residue of SRP54 protein was − 1.23 kcal/mol (Fig. 3B), also indicating the binding might be dominated by hydrophobic interactions.

As shown in Fig. 3E, the main chain amino group N-H of Leu19 in the IL-6’s signal peptide formed a hydrogen bond with side chain carboxyl group O of Asp313 residue in the SRP54 subunit, in a bond length of 2.9 Å. Analysis of the Interface energy module revealed that the paired energy between the Leu19 of IL-6’s signal peptide and the Asp313 residue of SRP54 protein was − 5.39 kcal/mol, also indicating the binding might be dominated by hydrophobic interactions.

By using Rosetta’s Interface analyzer module analysis, it was found that the binding free energy between the SRP54M subunit and signal peptides were − 37.06, − 40.59 and − 46.35 kcal/mol for origin, tPA, and IL-6, respectively. The results showed IL-6’s signal peptide could bind better than signal peptide of tPA and origin, subsequentially.

Then the translation levels of the three mRNAs encoding RBD linking different signal sequences (origin, tPA, IL-6) in 293T and DC2.4 cells were tested. IL-6 consistently showed the highest level of RBD expression, surpassing both tPA and the origin in 293T cells and their supernatants (Fig. 3F). A similar trend was observed in RBD expression in DC2.4 cells and their supernatants (Fig. 3G). These results suggested that stronger binding between the signal peptide and the SRP54M subunit led to enhanced antigen expression.

### Signal sequence-modified mRNA vaccines induced different antibody titers and different response of memory B cells against virus

**Fig. 4:**
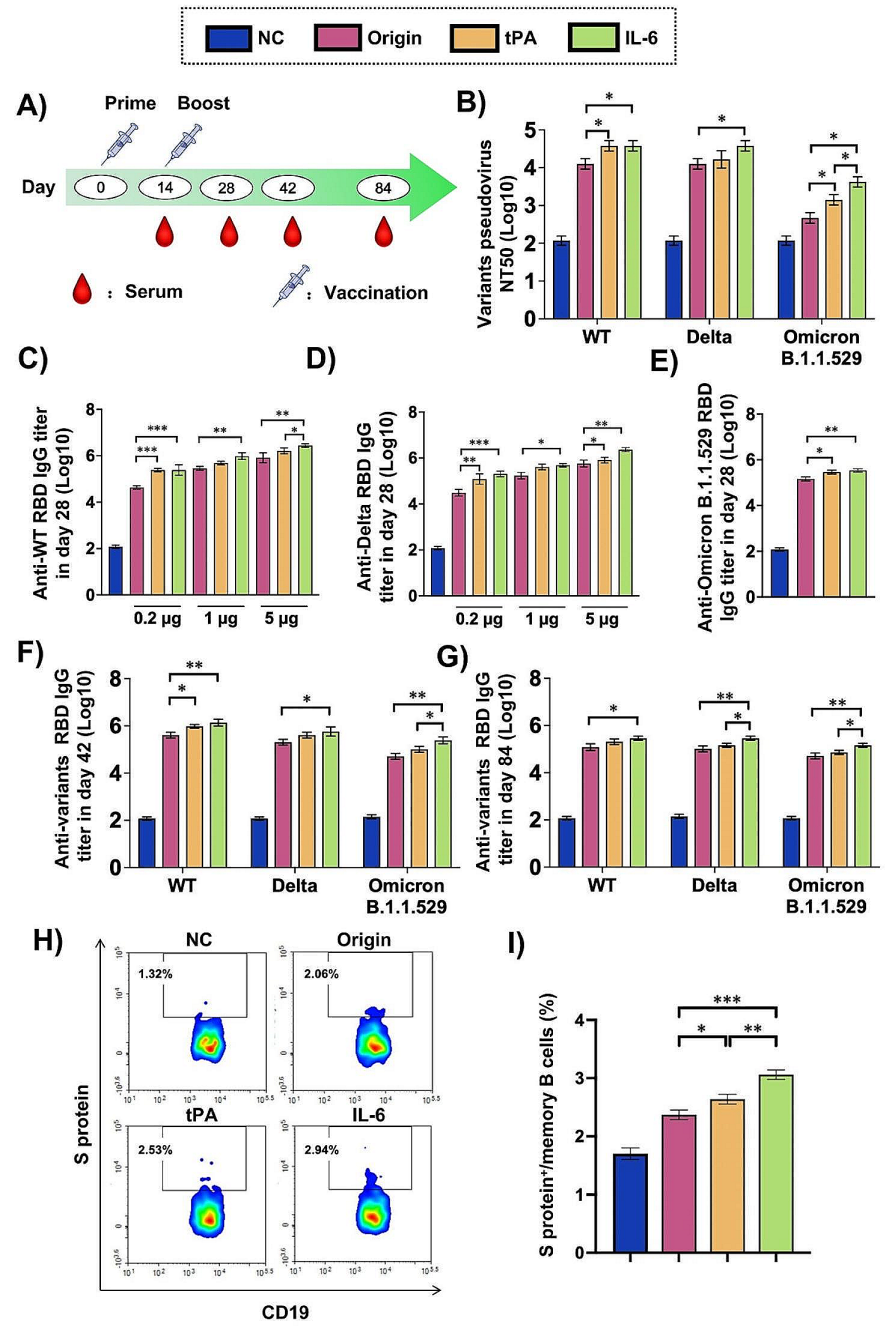
The specific humoral immune response elicited by mRNA vaccines with various signal sequence modifications against different SARS-CoV-2 variants. (**A**) The arrangement of the mouse experiment was diagrammed. (**B**) Pseudovirus neutralization assays for the WT, Delta, and Omicron B.1.1.529 variants were conducted on day 42. (**C**-**E**) Serum ELISA quantified RBD^WT^, RBD^Delta^ and RBD^Omicron^-specific IgG titers on day 28. Additionally, ELISA assessed the variants’ RBD-specific IgG titers in serum at day (**F**) 42 and (**G**) 84 following administration of RBD^WT^ mRNA vaccines with different signal sequences. (**H**) Representative flow cytometry diagrams and (**I**) quantification of S protein-specific memory B cells in spleen lymphocytes were provided for day 84 (*n* = 4). The gating strategy was depicted in Figure S3. The data were shown as mean ± SEM, *n* = 4. ^*^P < 0.05, ^**^P < 0.01, ^***^P < 0.001

We further tested the humoral immune activation effect of the vaccines on SARS-CoV-2 in vivo. The sequence designs of the three different signal sequences connected RBD^WT^ mRNA vaccines were showed in Fig. 4A. 0.2, 1, 5 µg mRNA vaccines were injected to the leg of BALB/c mice at day 0 and 14, and serum sampling at day 14, 28, 42, and 84 were conducted (Fig. 4B). The tPA and IL-6 sequentially presented higher anti-WT, Delta and Omicron RBD IgG titers than origin in day 14 and 28. These effects were in a dose-dependent manner (Figure S2, 4 C-E). At the day of 42 and 84, serum IgG titers of the three strains all decreased, but the trend remained unchanged (Fig. 4F, G). At the same time, we tested the neutralizing antibody titers of the three strains in day 42, and we could see matching results (Fig. 4B).

As is known, memory B cells play a significant role to help the body fight against SAS-CoV-2. We used gating strategy of Figure S3 to define S-2P specific memory B cells. We could see the S protein specific memory B cells of IL-6, tPA was sequentially higher than that of origin (Fig. 4H, I).

The results revealed that the signal sequences of tPA and IL-6 linked RBD^WT^ mRNA vaccines induced better humoral immunity than that of origin, sequentially.

### Signal sequence-modified mRNA vaccines induced different response of T cells against virus

**Fig. 5:**
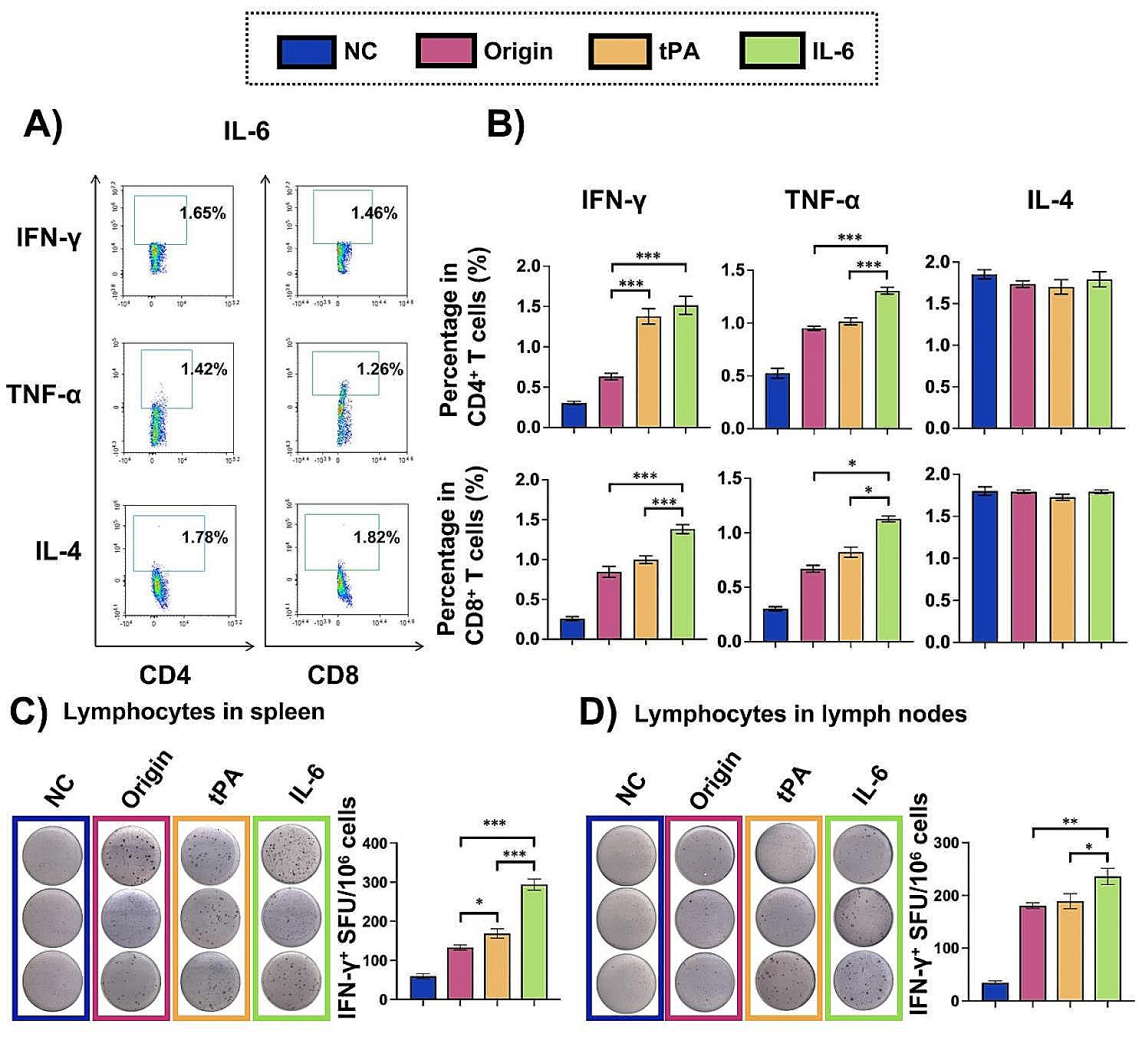
T cell response of mRNA vaccines modified with different signal sequences. Lymphocytes from the spleen and lymph nodes were collected on day 84, as outlined in Fig. 4A, and subsequently stimulated with SARS-CoV-2 S protein peptide pools. (**A**) Representative flow cytometry diagrams of IL-6 group and (**B**) quantifications of intracellular cytokines in CD4^+^ and CD8^+^ memory T cells from the spleen (*n* = 4) were provided. The gating strategy was detailed in Figure S4. ELISpot assays were conducted to determine the presence of IFN-γ^+^ T cells in (**C**) spleens and (**D**) lymph nodes (*n* = 3), with images and quantitative data shown respectively. The results were presented as the mean ± SEM, *n* = 3. ^*^P < 0.05, ^**^P < 0.01, ^***^P < 0.001

As we know, B cells response was supported by Th cells, and Tc cells could directly kill virus-infected cells. We employed flow cytometry to detect IFN-γ and TNF-α secreting memory Th/Tc cells, and IL-4 secreting memory Th/Tc cells within CD44 ^high^CD4^+^/CD8^+^ cells (Figure S4). The results indicated that memory Th1 and Tc1 cells in response to tPA and IL-6 modified mRNA vaccines were more effective than those responding to the original signal sequence, while memory Th2 and Tc2 cells showed no significant differences (Fig. 5A, B). The ability of lymphocytes in the spleen and lymph nodes to secrete IFN-γ was then assessed using an ELISpot assay. Representative images and statistical histograms (Fig. 5C, D) demonstrated that lymphocytes in the IL-6 group secreted more IFN-γ than those in the tPA and origin groups, sequentially. These findings suggest that tPA and IL-6 linked RBD^WT^ mRNA vaccines sequentially induced stronger Th1/Tc1 biased memory T cell reaction compared to the origin. Additionally, the results corresponded with the B cell responses observed earlier.

### Safety profiles of mRNA vaccines with different signal sequences

**Fig. 6:**
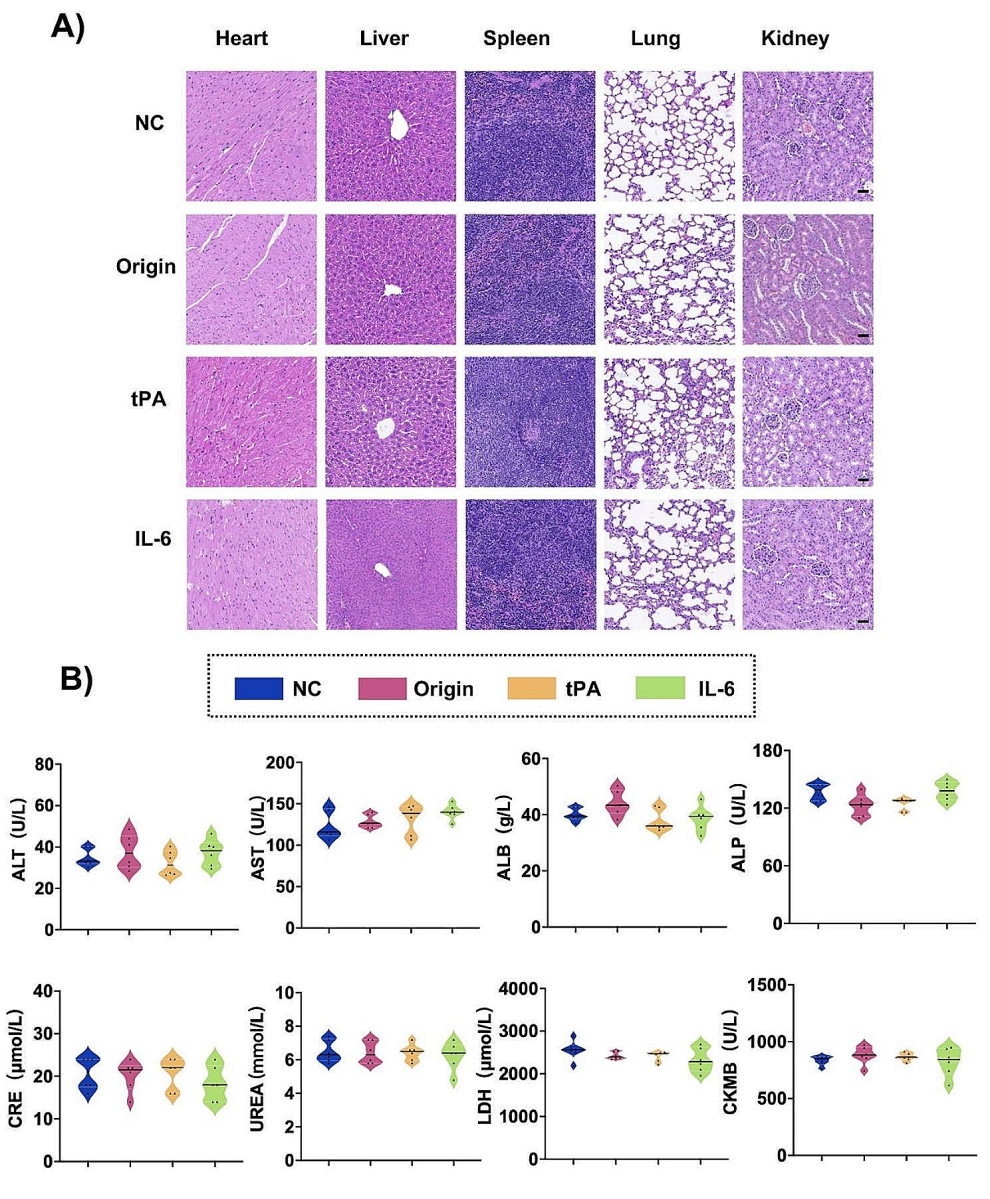
Safety assessment of mRNA vaccines with different signal sequence modifications. (**A**) HE staining (scale bar = 50 μm) and (**B**) serum biochemical examination by 5 µg mRNA vaccines intramuscular injected to BALB/c mice at day 84 (*n* = 6). The data were presented as mean of black solid line ± quartiles of white dotted lines (violin plot)

Both the efficacy and safety of the vaccine are indispensable. As showed in Fig. 6A, pathological examinations of the heart, liver, spleen, lung, and kidney across all groups revealed no indicators of safety concerns. Furthermore, blood biochemical tests, including measurements of ALT, AST, ALB, and ALP for liver function, CRE and UREA for kidney function, and LDH and CKMB for heart function, showed no significant changes across the groups (Fig. 6B). In conclusion, mRNA vaccines with varying signal sequences were found to be relatively safe.

## Discussion

mRNA vaccines have demonstrated their potent and positive role in combating infectious diseases and tumors. In developing our mRNA vaccine system against SARS-CoV-2, we selected MIC1 as the ionizable lipid material, combined with other components, to encapsulate mRNAs encoding antigens. Signal sequences from tPA and IL-6 sequentially showed more enhanced antigen expression in RBD^WT^ mRNA vaccines than the original signal sequence in vitro. These findings aligned with computational simulations indicating that tPA and IL-6’s signal peptides bound more effectively to the SRP54M subunit than the original signal peptide, sequentially. Furthermore, we found that signal sequences from IL-6 and tPA sequentially elicited higher binding and neutralizing antibody titers against SARS-CoV-2 in vivo, surpassing the original signal peptide. This enhanced response might be attributed to their superior stimulation of Th1/Tc1 biased memory T cell and memory B cell responses. Importantly, all signal sequence-modified RBD mRNA vaccines in our study exhibited good administration safety.

In the realm of clinical trials, several mRNA vaccines have been developed based on the RBD of SARS-CoV-2 [12, 39]. However, challenges arise due to the limited size of RBD monomers and their suboptimal immunogenicity, which hinder effective recognition by the immune system [17, 40]. Moreover, antigens derived from the WT strain of SARS-CoV-2 have demonstrated decreased binding and neutralization antibody titers against various variant strains [41]. Notwithstanding these obstacles, our study revealed that even against the Delta and Omicron variants, which exhibit relatively lower protective efficacy, the antigens of the RBD^WT^, when linked with enhanced signal sequences, provided improved protection. The efficacy of these modified RBD^WT^ antigens with improved signal sequences against variant strains was found to be on par with that of antigens based on the original signal sequence and the RBD^WT^ against their corresponding WT strain, as delineated in Fig. 4B-G and S2.

The artificially designed signal sequences were also employed to enhance protein expression in vitro. However, their application in vivo might have been limited due to potential incompatibility with natural bodily processes. We undertook a comprehensive comparison of natural signal sequences from IL-6 and tPA, which are widely utilized in protein production and gene vaccines. tPA signal sequence is one of the most widely used signal sequences when using eukaryotic and prokaryotic cells to produce proteins. Some researchers also have constructed DNA vaccines expressing tuberculosis antigens with the tPA signal sequence to replace original signal [42, 43]. Importantly, tPA signal sequence has also been used to construct protein vaccine and adenoviral vector vaccines against virus [12]. Moreover, signal sequence of interleukin (IL)-6 is classical in the realm of protein expression [44]. Despite their prevalent use, there was a lack of research regarding their comparative efficacy in mRNA vaccines.

The initial scanning and recognition of the signal sequence by the SRP were critical steps in directing the translating ribosome towards the ER [45–47]. Additionally, the stability of the SRP-ribosome nascent chain (RNC) complex, particularly after its conformational change, together with the interaction of this complex with the translocon post-transformation, might played significant roles in modulating the efficiency of mRNA translation [48]. Our research established a notable positive correlation between the affinity of the signal peptide for the SRP54M subunit and the translation efficiency of the antigen protein. This correlation can probably extend to influence followed immune responses. These findings contribute substantially to our understanding of the mechanisms underlying mRNA vaccine efficacy.

## Conclusions

In conclusion, our study found that substituting the signal sequence in mRNA vaccine formulations constituted a viable approach for amplifying the immune response against viral infection. The signal sequences derived from IL-6 and tPA exhibited enhanced immune-stimulatory effects in comparison to the original virus antigen. This increased efficacy may be attributed to the higher affinity of IL-6 and tPA for the SRP54M subunit, which potentially impacts the translation process of the mRNAs. Our findings provide an initial framework that could guide the continued development and refinement of mRNA vaccines aimed at a broad spectrum of viral infection.

## Materials and methods

### In vitro transcription of mRNA

mRNA was transcribed in vitro from a linearized DNA template. This template encompassed LUC (Luciferase, GenBank: Q27758), the wildtype RBD from the Spike (S) protein of SARS-CoV-2 (GenBank: P0DTC2), and signal sequences of origin (GenBank: P0DTC2), IL-6 (GenBank: P08505), and tPA (GenBank: P11214). The transcription process employed T7 RNA polymerase (Vazyme Biotech Co., Ltd).

### Preparation and characterization of mRNA vaccines

Our team previously reported the synthesis steps for MIC1 lipids [15]. LNPs for mRNA vaccines, encapsulating mRNA encoding LUC and mRNA encoding RBD linked with various signal sequences, were prepared using a microfluidic device (Micro&Nano Biologics). Initially, MIC1, DSPC, cholesterol, and DMG-PEG2k were dissolved in ethanol at a molar ratio of 35:16:46.5:2.5, forming the organic phase [49–51]. Concurrently, mRNA was dissolved in citric acid buffer (pH 6.0) to form the aqueous phase. The mRNA vaccines were then produced by mixing these organic and aqueous phases. Subsequently, ethanol was removed through ultrafiltration.

The particle size and zeta potential of the mRNA LNPs were determined using a Zetasizer Nano ZS90 (Malvern). Encapsulation efficiency was calculated as [(1 - m_free_/m_total_) × 100%], following our previously established methodology [52]. The morphology of the mRNA vaccines was visualized using 2% phosphotungstic acid staining, observed under a TEM FEI Talos F200XG2 AEMC (Thermo Fisher).

### Computational simulation

The structural simulation of the SRP54M subunit and signal peptides utilized Alphafold2. The optimal model was selected based on Alphafold2 scoring for subsequent docking analyses. Initial rigid docking was conducted on the HPEPDOCK online platform to acquire the preliminary conformation between the protein and signal peptides.

Following the rigid docking results, flexible docking was performed using Rosetta Flexpepdock. Result selection was based on Rosetta’s built-in score module for screening purposes. Ligplot software aided in analyzing the binding interactions between the molecules. Interface analyzer and Interface energy modules from Rosetta were employed to evaluate the overall interaction binding energy between the signal peptides and the SRP54M subunit, as well as the binding energy of key amino acids. The docking conformation of the signal peptides with the SRP54M subunit was visualized using PyMOL software.

### Cell Counting Kit-8 (CCK-8) assay

The assay was conducted by following the instructions of the kit (Yeasen, Cat: 40203es60). After seeding 293T or DC2.4 cells (100 uL) in a 96 well plate with 5 × 10^3^/well, the plate was cultured for 24 h. 0, 0.2, 0.4, 0.8, 1.2, 1.6, 2, and 2.4 µg LUC mRNA LNPs was added to each well and cultured for another 24 h. Then, 10 µL CCK-8 solution was added to each well and cultured for 4 h. The absorbance (A) at 450 nm was measured using a microplate reader (Perkin Elmer). Cell viability (%) was calculated by [(A_drugs_ - A_blank_) / (A_cells_ - A_blank_) × 100%].

### Animal treatment

Animals, procured from SPF (Beijing) Biotechnology Co., Ltd., were housed under controlled conditions with a 12-hour light/dark cycle at a temperature of 22 ± 2 °C. They had unrestricted access to sterilized food and water. The animal studies adhered to the regulations of experimental animal administration as mandated by the Experimental Animal Ethics Committee of West China Hospital, Sichuan University, with the ethical filing number of 20,221,110,006.

Male BALB/c mice, weighing 18–20 g, were administered 20 µg of LUC mRNA-containing LNPs via intramuscular injection. Six hours post-injection, 150 mg/kg D-luciferin potassium salt (Yeasen) was injected intraperitoneally into the mice. Ten minutes following this injection, the bioluminescent imaging of the body was conducted using the IVIS Lumina system (Perkin Elmer).

BALB/c mice, each weighing between 18 and 20 g, were randomly assigned into six groups, with 4 or 6 mice per group. The mice underwent two rounds of intramuscular immunizations with low (0.2 µg), medium (1 µg), and high (5 µg) doses of mRNA vaccines on day 0 and day 14. A control group received equivalent volumes of the vehicle as a normal control (NC). Serum samples were collected on days 14, 28, 42, and 84. On day 84, selected tissues were harvested to assess B and T cell responses and to evaluate the safety of the vaccine formulations. In another experimental set-up, a dose of 5 µg of mRNA vaccines was administered on days 0 and 14. Serum samples were collected on days 14, 28, 42, 56, 70, and 84 post-vaccination. For the vaccine formulations stored at 4 °C for 14 and 28 days, serum samples were collected on day 28 following the initial vaccination.

### Enzyme-linked immunosorbent assay (ELISA)

High binding polystyrene plates (Corning) were coated with the RBD of SARS-CoV-2 S protein variants, including the 2019-nCoV (WT), B.1.617.2 (Delta), and Omicron B.1.1.529. Following overnight incubation at 4 °C, the plates were blocked using 2% BSA. Serum samples, inactivated at 56 °C for 30 min, underwent two-fold serial dilution before being added to the plates, and then incubated overnight at 4 °C. Horseradish peroxidase (HRP)-conjugated anti-mouse IgG was subsequently applied and allowed to incubate for 2 h at room temperature (25 °C). Following washing steps, Tetramethylbenzidine (TMB) substrate (Solarbio) was added. The enzymatic reaction was terminated with H_2_SO_4_, and absorbance was measured at 450 nm using a microplate reader (Perkin Elmer). The endpoint titer was determined as the serum dilution that exceeded control values.

293T and DC2.4 cells were seeded in 24-well plates (2 × 10^5^ cells/well) and cultured for 24 h. These cells were then treated with 1 µg of different mRNAs, complexed with Lipofectamine 2000 (Invitrogen) as the transfection reagent. RBD protein expression in both cells and supernatant was quantified using the SARS-CoV-2 RBD Detection ELISA Kit (Vazyme Biotech Co., Ltd), with calculations based on indirect measurement of RBD expression.

### Pseudovirus neutralization assay

Neutralization assays were conducted against SARS-CoV-2 pseudotyped viruses [2019-nCoV (WT), B.1.617.2 (Delta), Omicron B.1.1.529] carrying GFP-LUC (Genomeditech) to determine their respective neutralization titers. The pseudoviruses were initially diluted in complete medium. Subsequently, three-fold serial dilutions of serum samples were added and incubated for one hour at 37 °C. Thereafter, 293T-hACE2 cells were introduced into each well, followed by a further incubation at 37 °C for 48 h. Enzyme substrate was added to quantify LUC activity using a microplate reader (Perkin Elmer). The neutralization endpoint, NT50, was identified as the serum dilution required to achieve 50% inhibition of LUC activity compared to virus control samples.

### T cell flow cytometry

Antigen-specific memory CD4^+^ and CD8^+^ T cell immune responses were assessed using an intracellular cytokine staining (ICS) assay [53, 54]. Briefly, freshly isolated splenocytes from the NC and mRNA vaccine-immunized groups on day 42, along with SARS-CoV-2 S protein peptide pools (2 µg/ml for each peptide), were cultured in 24-well plates at a density of 2 × 10^6^ cells/well. After a 2-hour incubation, monensin (YEASEN) was added to each well to inhibit cytokine secretion. Twelve hours later, the cells were harvested and stained for 40 min with PE anti-mouse CD4, PerCP anti-mouse CD8, Alexa Fluor 700 anti-mouse MHC II, BV421 anti-mouse B220, BV510 anti-mouse CD44, BV711 anti-mouse CD3 (Biolegend), and Pacific Orange for live/dead cells (Thermo Fisher). Subsequently, the cells were fixed with fixation buffer for 20 min and permeabilized in 1× permeabilization buffer (Biolegend). Intracellular staining was performed using FITC anti-mouse IFN-γ, PE/Cyanine7 anti-mouse TNF-α (Bioss), and APC anti-mouse IL-4 (Biolegend). Following three washes with PBS, the cells were analyzed by flow cytometry using a NovoCyteTM system (Eisen).

### ELISpot assay

To evaluate antigen-specific T cell responses, an IFN-γ ELISpot assay was performed (Mabtech). Freshly isolated splenocytes (5 × 10^5^ cells/well) from vaccinated mice on day 84, along with SARS-CoV-2 S protein peptide pools (2 µg/ml for each peptide), were seeded into the assay plates. Unstimulated cells served as negative controls. The plates were incubated at 37 °C in a 5% CO_2_ atmosphere for 36 h. Following incubation, cells were removed, and the plates were washed five times with 200 µL of PBS per well. Subsequent steps were conducted according to the kit’s instructions.

### B cell flow cytometry

The kinetics of S-specific memory B cell responses were determined according to previously described methods [55, 56]. Freshly isolated splenocytes from the NC group and five mRNA vaccine-immunized groups at day 84 were initially stained with AVI tag-labeled S protein (Vazyme) for 30 min at 4 °C. After three washes with PBS, the cells underwent further staining with FITC anti-mouse CD16, PE anti-mouse CD4, PE/Cyanine5-conjugated streptavidin (binding to the AVI tag), PerCP/Cyanine5.5 anti-mouse IgM, PE/Cyanine7 anti-mouse CD20, APC/Cyanine7 anti-mouse CD14, Pacific Blue anti-mouse CD19, Qdot655 anti-mouse IgD, Qdot705 anti-mouse CD3 (Biolegend), and Pacific Orange for live/dead cells (Thermo Fisher). Subsequent to three additional PBS washes, the cells were analyzed using NovoCyteTM flow cytometry (Eisen).

### Safety assessment

To evaluate the safety of the mRNA vaccines, biochemical analysis of end-point serum samples was conducted. Serum samples collected on day 84 were analyzed for levels of ALT, AST, ALB, ALP, CRE, UREA, LDH and CKMB using an automatic hematological biochemical analyzer (Hitachi). Concurrently, organs from each mouse were excised for hematoxylin and eosin (H&E) staining. All slide images were captured using an Olympus-BX 43 fluorescence microscope (Olympus).

### Quantification and statistical analysis

Statistical analysis was conducted using one-way analysis of variance (ANOVA). Data are presented as mean ± SEM or mean ± quartiles. Differences were considered statistically significant at *P* < 0.05. All analyses were performed using SPSS software, version 26.0.

### Electronic supplementary material

Below is the link to the electronic supplementary material.

Supplementary Material 1

## Data Availability

No datasets were generated or analysed during the current study.
