# Supplementary material for: Efficient signal sequence of mRNA vaccines enhances the antigen expression to expand the immune protection against viral infection
Source: J Nanobiotechnology. 2024 May 28;22:295. doi: 10.1186/s12951-024-02488-3 (PMC11134928; doi:10.1186/s12951-024-02488-3)
Supplement: Supplementary file 1 — Supplementary Material 1 [file 12951_2024_2488_MOESM1_ESM.docx]

**Supplementary figures**

**
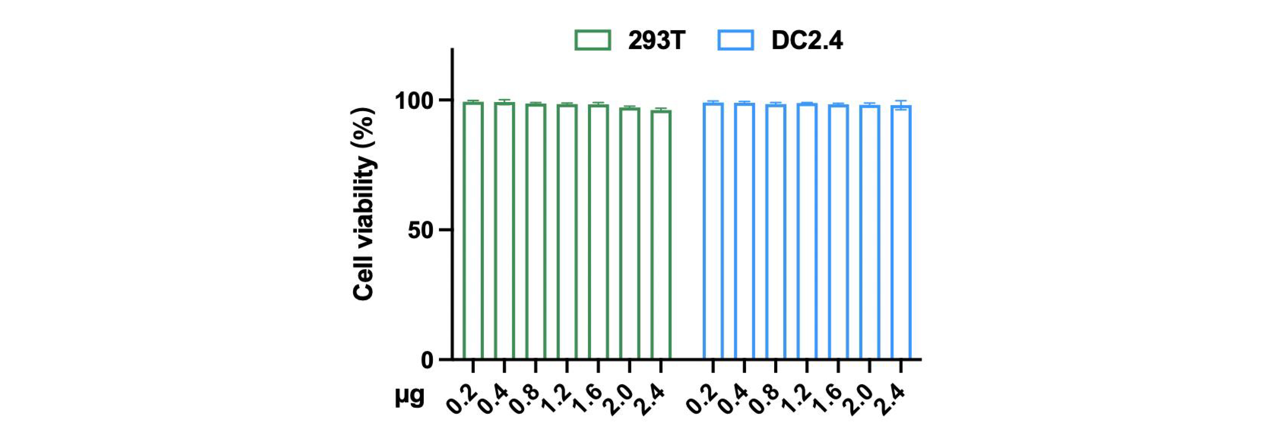
**

**Figure S1.** Cytotoxicity of mRNA LNPs in 293T and DC2.4 cells. Cell viability of 0, 0.2, 0.4, 0.8, 1.2, 1.6, 2, and 2.4 μg LUC mRNA LNPs for 293T and DC2.4 cells were measured by CCK-8 assay. The data were shown as mean ± SEM, n = 3.

**
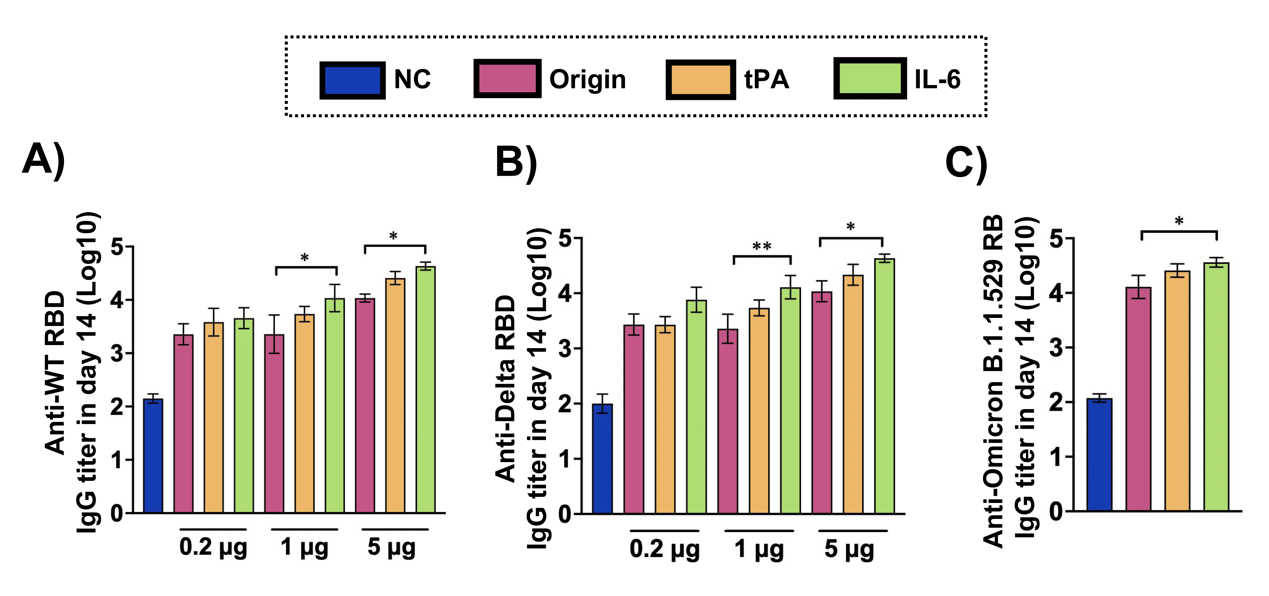
Figure S2.** Serum ELISA of (A) RBD^WT^ and (B) RBD^Delta^-specific IgG titers of 0.2, 1 and 5 μg RBD^WT^ mRNA vaccines with different signal sequences at day 14. (C) Serum ELISA of RBD^Omicron^-specific IgG titers of 5 μg RBD^WT^ mRNA vaccines with different signal sequences at day 14. The data were shown as mean ± SEM, n = 4. ^*^*P* < 0.05, ^**^*P* < 0.01.

**
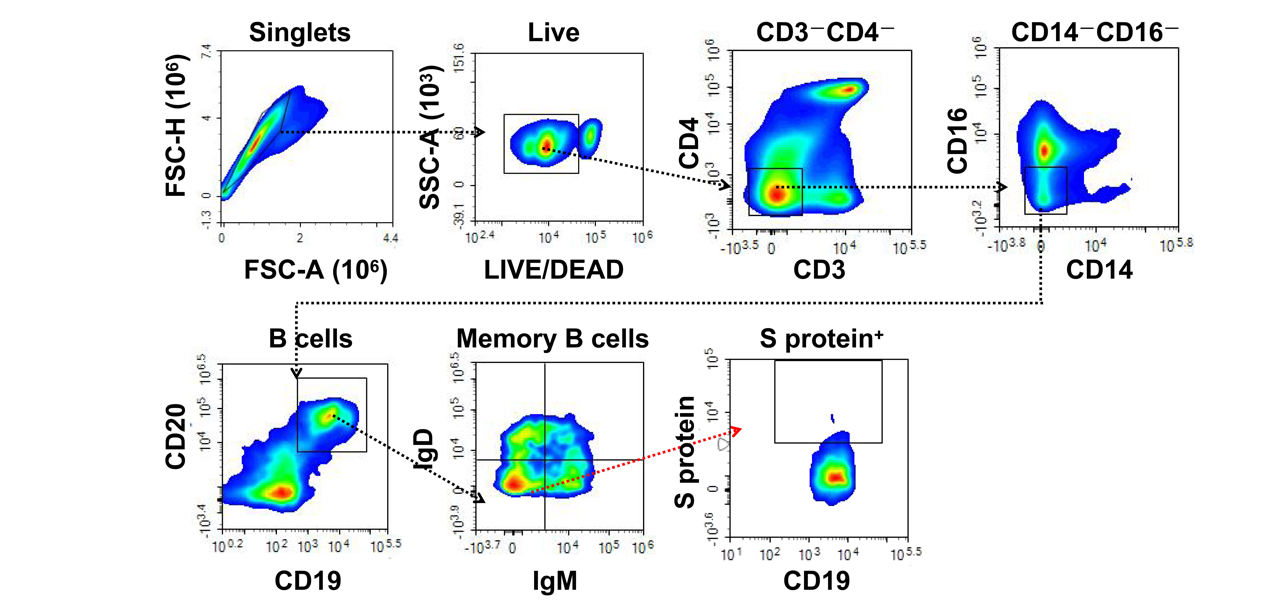
Figure S3.** B cell gating strategy (related to Figure 3 H, I). Cells were gated as singlets and live cells on forward and side scatter and a live/dead stain. CD3^－^, CD4^－^ cells were then gated on absence of CD14 and CD16 expression and positive expression of CD20 and CD19. Memory B cells were selected based on lack of IgD or IgM. Finally, S-2P probes were used to determine binding specificity.

**
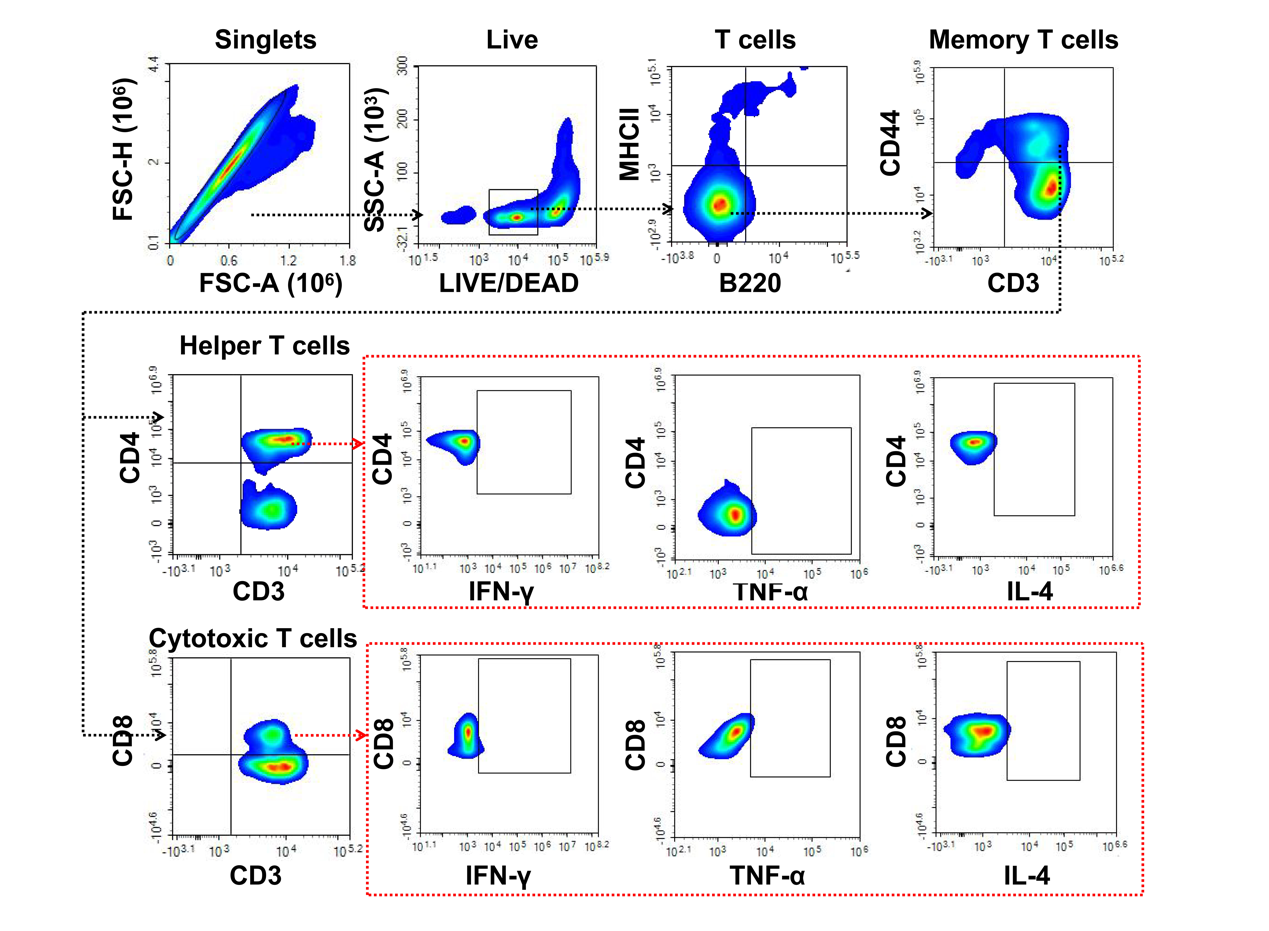
Figure S4.** T cell gating strategy (related to Figure 4 A, B). Cells were gated as singlets and live cells on forward and side scatter and a live/dead stain. CD3^+^ and CD44^+^ cells were selected on MHCII^－^ and B220^－^ cells. Th cells were gated by CD4^+^, and Tc cells were gated by CD8^+^. Finally, ICS of IFN-γ^+^, TNF-α^+^and IL-2^+^ cells in CD4^+^ and CD8^+^ cells, respectively, were used to determine memory T cell response.
